# The histone methyltransferase Mixed-lineage-leukemia-1 drives T cell phenotype via Notch signaling in diabetic tissue repair

**DOI:** 10.1172/jci.insight.179012

**Published:** 2024-10-08

**Authors:** William J. Melvin, Tyler M. Bauer, Kevin D. Mangum, Christopher O. Audu, James Shadiow, Emily C. Barrett, Amrita D. Joshi, Jadie Y. Moon, Rachael Bogle, Purba Mazumder, Sonya J. Wolf, Steven L. Kunke, Johann E. Gudjonsson, Frank M. Davis, Katherine A. Gallagher

**Affiliations:** 1 Section of Vascular Surgery, Department of Surgery,; 2 Department of Dermatology, and; 3 Department of Microbiology and Immunology, University of Michigan, Ann Arbor, Michigan, USA.

**Keywords:** Immunology, Diabetes, Epigenetics, T cells

## Abstract

Immune cell–mediated inflammation is important in normal tissue regeneration but can be pathologic in diabetic wounds. Limited literature exists on the role of CD4^+^ T cells in normal or diabetic wound repair; however, the imbalance of CD4^+^ Th17/Tregs has been found to promote inflammation in other diabetic tissues. Here, using human tissue and murine transgenic models, we identified that the histone methyltransferase Mixed-lineage-leukemia-1 (MLL1) directly regulates the Th17 transcription factor RORγ via an H3K4me3 mechanism and increases expression of Notch receptors and downstream Notch signaling. Furthermore, we found that Notch receptor signaling regulates CD4^+^ Th cell differentiation and is critical for normal wound repair, and loss of upstream Notch pathway mediators or receptors in CD4^+^ T cells resulted in the loss of CD4^+^ Th cell differentiation in wounds. In diabetes, MLL1 and Notch-receptor signaling was upregulated in wound CD4^+^ Th cells, driving CD4^+^ T cells toward the Th17 cell phenotype. Treatment of diabetic wound CD4^+^ T cells with a small molecule inhibitor of MLL1 (MI-2) yielded a significant reduction in CD4^+^ Th17 cells and IL-17A. This is the first study to our knowledge to identify the MLL1-mediated mechanisms responsible for regulating the Th17/Treg balance in normal and diabetic wounds and to define the complex role of Notch signaling in CD4^+^ T cells in wounds, where increased or decreased Notch signaling both result in pathologic wound repair. Therapeutic targeting of MLL1 in diabetic CD4^+^ Th cells may decrease pathologic inflammation through regulation of CD4^+^ T cell differentiation.

## Abstract 2

MLL1, a histone methyltransferase, regulates T-cell differentiation in diabetic wounds, causing pathologic inflammation, and may serve as a therapeutic target to improve diabetic wound healing.

## Graphical abstract

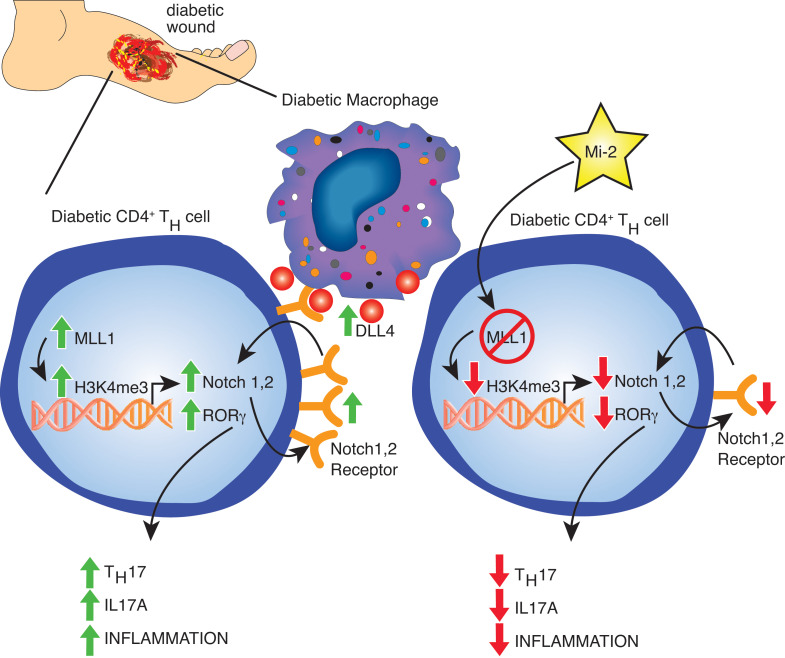

## Introduction

A deliberate and orchestrated response must occur between immune and structural cells to promote optimal tissue repair and regeneration. Pathologic states where tissue repair is impaired are characterized by dysregulated responses in immune cells and/or structural cells. In the setting of diabetes, chronic wounds are in a persistent inflammatory state, unable to transition out of the inflammatory phase to the proliferative phase of wound repair (1–4). The etiology of these pathologic changes in diabetic immune cells are multifactorial and not completely understood, but epigenetic alterations that occur in the diabetic condition have been shown to control Mφ phenotypes in diabetic wound tissue (2, 5–7).

Although not as well studied in the setting of wound repair, CD4^+^ Th cells are classically thought to play a supportive role in wound healing, influencing other key immune and structural cells present in the wound, and guiding these cells through the phases of wound healing in a regulated fashion (8–11). Specifically, circulating CD4^+^ T cells that infiltrate into the wound tissue following injury are the primary driver of CD4^+^ T cell dynamics in wound repair (9, 12, 13). Existing studies are limited but have demonstrated that depletion of CD4^+^ Th cells at days 5–7 in murine wounds decreases wound inflammation and impairs wound healing, which established a critical role of CD4^+^ Th cell in orchestrating tissue repair processes (10, 13–16). The balance of 2 particular CD4^+^ phenotypes, the antiinflammatory Tregs and proinflammatory IL-17A–producing CD4^+^ Th17 cells, can independently dictate inflammation in tissues (17, 18) and can be relevant to complications of diabetes, by which the chronic inflammatory state is often hallmarked with high proportions of Th17 differentiation (19–21).

Despite a well-documented understanding of CD4^+^ Th cells and their role in inflammation in many tissues/disease states, surprisingly little is known about these cells in the setting of wound repair, specifically in diabetic wound healing. In the setting of nonpathologic wound repair, Tregs have been shown to prevent proinflammatory Mφ accumulation, and Treg-specific ablation has been shown to delay wound closure in normal mice, but Tregs have not been well studied in diabetic wound tissue (12, 22). It is known that diabetic wounds are characterized by delayed, prolonged inflammation, driven in part by high levels of IL-17A secreted in part by Th17 cells (23, 24). The mechanism that controls this imbalance in Th cell subtypes that is often seen in diabetic tissues is not understood.

The fate of Th cell differentiation in tissue is determined via its T cell receptor and cytokine signals (18); however, cell-to-cell Notch signaling is also known to play a critical role (25–27). In humans and mice, there are 4 Notch receptors (Notch 1–4) and 5 ligands (Delta-like 1, 3, 4, and Jagged 1 and 2). Once bound by its ligand, Notch receptors undergo proteolytic cleavage events that result in the release of Notch intracellular domain (NICD), which translocates to the nucleus and functions as a transcription factor to complex with the DNA binding protein CSL, Mastermind-like protein 1 (MAML), and histone acetyltransferases. This, in turn, promotes transcription of downstream Notch target genes (i.e., *Hes*, *Hey*). Depending on the context and location, loss of Notch signaling has been shown to both inhibit Th17 differentiation (27–31) and drive Treg differentiation (32, 33). In other instances, Notch signaling protects Tregs from apoptosis and enhances Treg functionality (34–36). These studies, which were primarily done in vitro, do not provide a succinct likely mechanism of how Notch receptor–mediated signaling influences CD4^+^ Th cell phenotypes in wound repair. Furthermore, although Notch signaling has been shown to be important in keratinocyte (37) and Mφ (7) functions in diabetic wounds, Notch signaling has not been studied in T cells in diabetic wounds. Given the prior literature examining other diabetic tissues, we theorized that increased Notch signaling in diabetic wound Th cells disrupts a physiologic balance between Th17 and Tregs.

Our group and others have found that epigenetic processes regulate immune cell phenotypes in various tissues/disease states. Mixed-lineage-leukemia-1 (MLL1) is a chromatin-modifying enzyme (CME) that upregulates gene transcription via trimethylation of the fourth lysine of the third histone (H3K4me3) at promoter sites (38) and has been shown to regulate Notch signaling pathways in oncogenesis research (39). The role of MLL1 in wound Th cells is unknown; however, in Mφs, it has been shown to drive dysregulated inflammation in diabetes (7). Furthermore, alterations to histone methylation by other CMEs have been shown to influence Th cell phenotype in vitro by directly altering key transcription factors for Th17 (RORγ) and Treg (FOXp3) differentiation (40, 41).

Here we show that Tregs and Th17 cells both play an important role in physiologic wound repair and that loss of Notch signaling impairs Th cell differentiation and subsequent healing. We found that MLL1 regulates expression of Notch1 and Notch2 receptors on wound Th cells and serves to increase Notch receptor–mediated signaling, and that loss of MLL1 in nondiabetic state impairs wound healing via loss of Th cell differentiation. In human and murine diabetic wounds, we found that Notch signaling is upregulated in Th cells and that the increased Notch signaling preferentially skews Th cell phenotype toward Th17. We identify that MLL1 is increased in diabetic CD4^+^ Th cells and that this increases the Th17 cell phenotype via upregulation of Notch receptor expression and direct regulation of the transcription factor associated with CD4^+^ Th17 cell differentiation, RORγ. Lastly, we show that MLL1 is a potential therapeutic target for nonhealing diabetic wounds, where pharmacologic inhibition of MLL1 reduces Th17 cells and IL-17A–based inflammation in the setting of diabetes.

## Results

### Human and murine diabetic wound CD4^+^ T cells display increased Th17 and IL17A.

**Figure 1:**
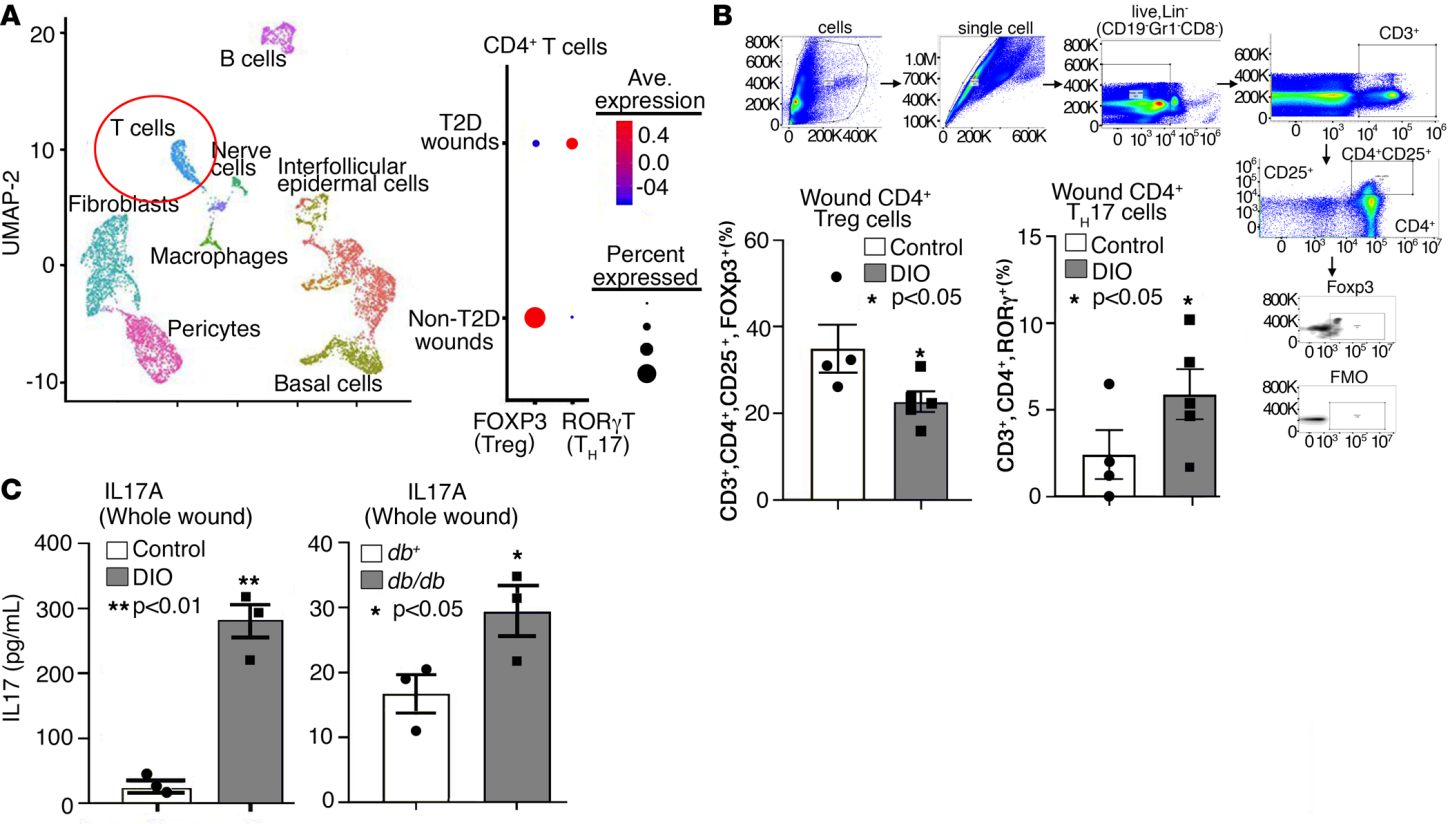
Human and murine diabetic wound CD4^+^ T cells display increased Th17 and IL-17A. (**A**) Cluster analysis UMAP of single-cell RNA-Seq from human T2D and non-T2D wounds showed 10 unique cell clusters (representative). Dot plot demonstrating increased Th17 and less Tregs in human diabetic wounds compared with nondiabetic wounds (*n* = 42). (**B**) Analytical flow cytometry of wound cell suspensions 5 days after wounding between ND (*n* = 4) and DIO (*n* = 5) mice. Tregs identified as Lineage^–^ (Lin^–^)/CD3^+^/CD4^+^/CD25^+^/FoxP3^+^, and Th17 cells identified as Lin^–^/CD3^+^/CD4^+^/RORγ^+^. For lineage-negative cells (defined as Lin^–^), we selected for cells that were negative for the following cell markers: CD19, GR-1, and CD8. (**C**) IL-17A measured by ELISA in wounds from ND (*n* = 4) and DIO (*n* = 4) mice, 5 days after wounding; and IL-17A levels measured by ELISA from whole wounds 5 days after wounding in *db^+^* (*n* = 3) and *db/db* (*n* = 3) mice. *P < 0.05, **P < 0.01. Data are presented as the mean ± SEM. All data are representative of 2–4 independent experiments. Data were first analyzed for normal distribution, and if data passed the normality test, 2-tailed Student’s *t* test was used.

Given that the balance of Treg/Th17 has been shown in diabetic retinopathy and nephropathy to modulate inflammation, and that Th17 cells have been shown to negatively influence wound repair (23, 24), we examined our human single-cell transcriptional profiling of diabetic and nondiabetic wounds to identify Treg/Th17 CD4^+^ Th cell phenotypes in wound tissue. This demonstrated increased CD4^+^ Th17 cells and decreased Tregs in diabetic wound tissue as compared with nondiabetic control wounds (Figure 1A). When we examined murine wound CD4^+^ Th cells isolated on day 5 after injury from diet-induced obesity (DIO), a murine model for diabetes, and normal diet control mice and performed intracellular flow cytometry (Supplemental Figure 1; supplemental material available online with this article; https://doi.org/10.1172/jci.insight.179012DS1), we found that DIO CD4^+^ wound T cells demonstrated an increased Th17 phenotype with fewer FoxP3^+^ Tregs (Figure 1B). Importantly, this imbalance between wound CD4^+^ Treg and Th17 phenotypes in diabetic wound tissue resulted in an increase in wound IL-17A as measured by ELISA in 2 murine models of diabetes (DIO and *db/db* mice) (Figure 1C).

### Notch signaling is increased in human and murine diabetic wound CD4^+^ Th cells.

**Figure 2:**
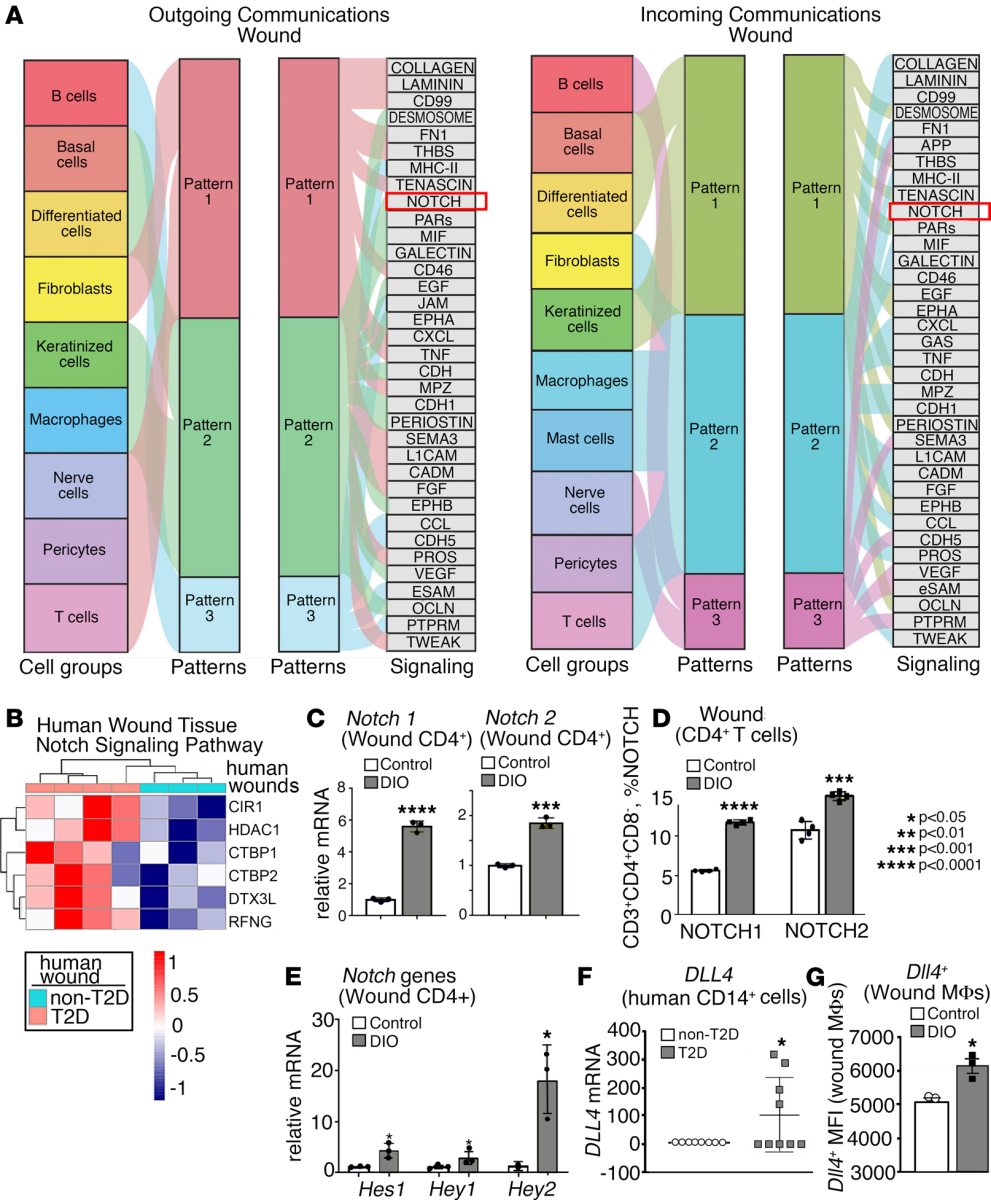
Notch signaling is increased in human and murine diabetic wound CD4^+^ Th cells. (**A**) River plots depicting cell groups and ligand-receptor pairs affect outgoing (signal source) and incoming (signal responder) pattern. The thickness of the flow indicates the contribution of the cell group or signaling pathway to each latent pattern (*n* = 10). (**B**) Human bulk RNA-Seq heatmap reflecting the expression profiles for selective genes (rows) across different samples (columns; stratified by T2D status) from Gene Ontology pathway analysis with upregulation of Notch signaling in T2D wounds compared with control wounds (*n* = 7). (**C**) *Notch* receptor expression in wound CD4^+^ Th cells 7 days after wounding between diet-induced obesity (DIO) mice and their normal diet, nondiabetic littermate controls (*n* = 6 mice/group, run in triplicate). (**D**) Analytical flow cytometry of wound cell suspensions 5 days after wounding between ND (*n* = 4) and DIO (*n* = 4) mice, evaluating for Lin^–^/CD3^+^/CD4^+^/Notch1^+^ (or Lin^–^/CD3^+^/CD4^+^/Notch2^+^). (**E**) *Hes1*, *Hey1*, and *Hey2* expression in wound CD4^+^ Th cells 5 days after wounding between ND and DIO mice (*n* = 6 mice/group, run in triplicate). (**F**) *DLL4* expression in peripheral human CD14^+^ monocytes between nondiabetic (*n* = 8) and diabetic (*n* = 9) donors. (**G**) Analytical flow cytometry between ND (*n* = 3) and DIO (*n* = 3) wound Mφs evaluating for Lin^−^/Ly6G^−^/CD11b^+^/DLL4^+^. *P < 0.05, ***P < 0.001, ****P < 0.0001. Data are presented as the mean ± SEM. Data were first analyzed for normal distribution, and if data passed the normality test, 2-tailed Student’s t test was used.

In order to define pathways that influence Th cell phenotypes, we analyzed our human skin wound single-cell RNA-Seq (scRNA-Seq) data for cell-cell interactions and the pathways involved in this signaling. Notch signaling was found to be substantially upregulated in diabetic wounds for both outgoing and incoming signaling pathways (Figure 2A). Given that our group and others have identified increased Notch ligands in Mφs from diabetic murine wounds (37, 42) and that Notch signaling plays an important role in regulating CD4^+^ Th cell phenotypes in various tissues, we hypothesized that Notch signaling may dictate CD4^+^ Th differentiation in both normal and pathological diabetic wound healing. First, to determine if Notch signaling is translationally relevant in human diabetic wounds, we examined RNA transcripts from human wounds; this identified increased Notch signaling transcripts in human diabetic wound tissue compared with nondiabetic controls (Figure 2B). Next, we intended to identify if our results in human tissue were similar in the physiologic murine model of diabetes, the DIO mouse model (2, 43). CD4^+^ Th cells that were isolated from wounds (day 7) of DIO and control mice demonstrated increased expression of Notch receptors *Notch1* and *Notch2* by quantitative PCR (qPCR) compared with nondiabetic littermate controls (Figure 2C). This correlated to increased Notch1 and Notch2 (but not Notch3 or Notch4) receptors expressed in CD4^+^ Th cells in DIO mice, compared with normal diet controls, as assessed by flow cytometry (Figure 2D). Additionally, downstream expression of Notch target genes *Hes1*, *Hey1*, and *Hey2* was increased in DIO wound CD4^+^ Th cells (Figure 2E), indicating actively increased Notch signaling in diabetic wound CD4^+^ Th cells.

The source of Notch ligands initiating receptor signaling in wound Th cells is likely multifactorial. Since Mφs are the primary antigen-presenting cell (APC) present in wound repair and dictate a number of pathologic processes in diabetic wounds related to inflammation, we examined their role in diabetic wounds in dictating Notch regulation of CD4^+^ Th cell phenotypes (44). Therefore, we isolated peripheral human CD14^+^ monocytes from patients with and without diabetes (sex and comorbid condition matched) and found an increase in the expression of Notch ligand *DLL4* in patients with diabetes compared with those without diabetes (Figure 2F). We also found an increase in the Notch ligand, *DLL4*, in DIO wound Mφs compared with nondiabetic littermate controls (Figure 2G). Our data suggest that there is increased Notch signaling in diabetic wound CD4^+^ Th cells via activation of *Notch1* and *Notch2* receptors on wound Th cells likely driven by increased DLL4 seen in diabetic Mφs.

### Notch signaling in cutaneous wounds regulates CD4^+^ Treg/ Th17 cell phenotypes.

**Figure 3:**
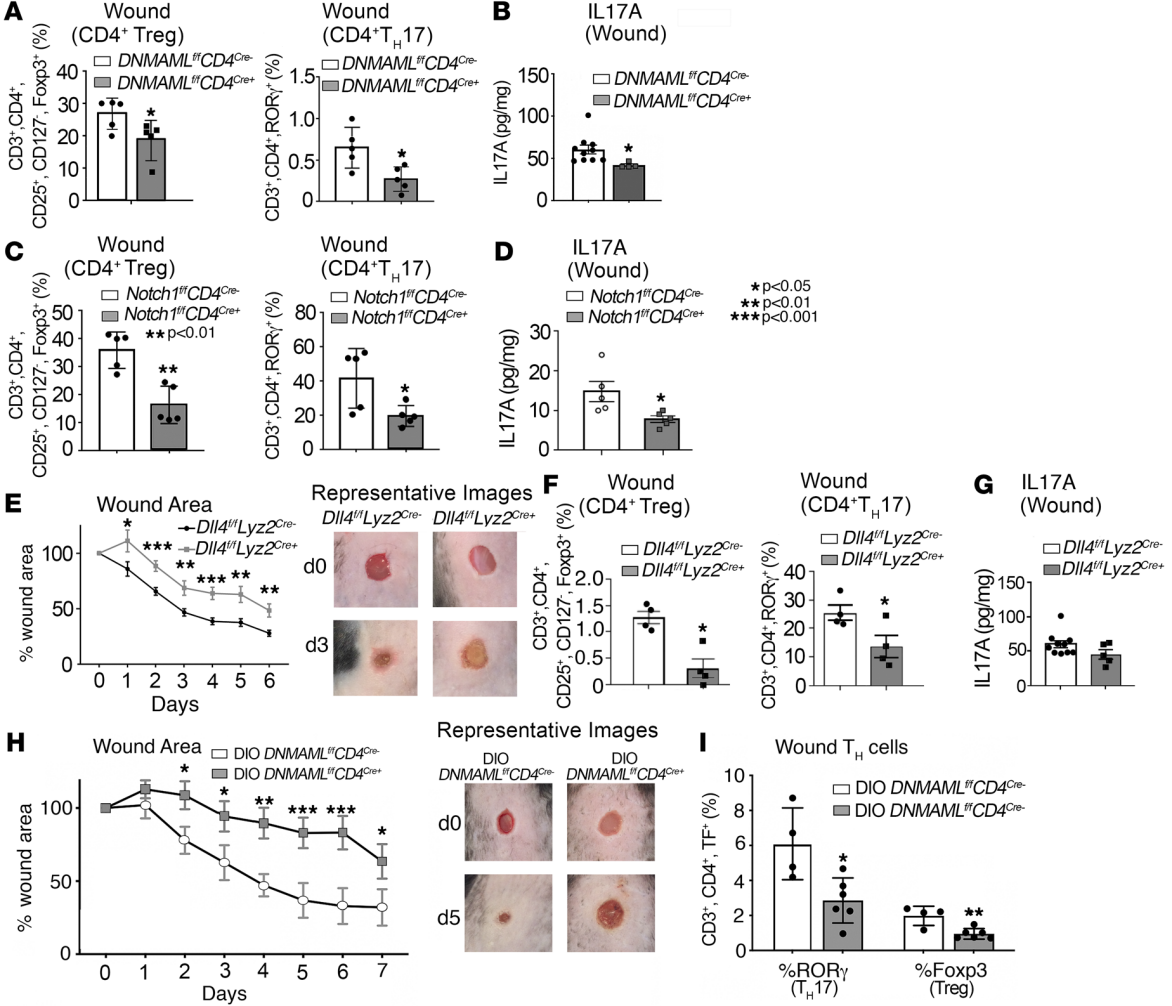
Depletion of Notch signaling prevents TH cell differentiation and worsens wound healing. (**A**) Analytical flow cytometry of cell suspensions 7 days after wounding between CD4^+^ Th cell–specific Notch-deficient diabetic (DIO) mice (DIO *DNMAML^fl/fl^ CD4^Cre+^)* (*n* = 5) and littermate controls (*n* = 5). Tregs identified as Lin^–^/CD3^+^/CD4^+^/CD25^+^/CD127^–^/FoxP3^+^ and Th17 cells identified as Lin^–^CD3^+^/CD4^+^/RORγ^+^. (**B**) IL-17 ELISA 5 days after wounding between CD4^+^ Th cell–specific Notch-deficient diabetic (DIO) mice (DIO *DNMAML^fl/fl^ CD4^Cre+^*) (*n* = 5) and littermate controls (*n* = 5). (**C**) Analytical flow cytometry of wound cell suspensions 5 days after wounding between CD4^+^ Th cell–specific Notch1 receptor–deficient mice (*Notch1^fl/fl^ CD4^cre+^*) (*n* = 5) and littermate controls (*n* = 5). Tregs identified as Lin^–^/CD3^+^/CD4^+^/CD25^+^/CD127^–^/FoxP3^+^, and Th17 cells identified as Lin^–^CD3^+^/CD4^+^/RORγ^+^. (**D**) IL-17 ELISA 5 days after wounding between CD4^+^ Th cell–specific Notch1 receptor–deficient mice (*Notch1^fl/fl^ CD4^cre+^*) (*n* = 5) and littermate controls (*n* = 5). (**E**) Wound healing curve for mice (*n* = 16) with myeloid cell–specific deficiency of DLL4 (*DLL4^fl/fl^ Lyz2^Cre+^*) compared with littermate controls (*n* = 14). (**F**) Analytical flow cytometry of wound cell suspensions 5 days after wounding between mice with myeloid cell–specific deficiency of DLL4 (*DLL4^fl/fl^ Lyz2^Cre+^*) (*n* = 4) and littermate controls (*n* = 4). Tregs identified as Lin^–^/CD3^+^/CD4^+^/CD25^+^/CD127^–^/FoxP3^+^, and TH17 cells identified as Lin^–^CD3^+^/CD4^+^/RORγ^+^. (**G**) IL-17 ELISA 5 days after wounding between mice with myeloid cell–specific deficiency of DLL4 (*DLL4^fl/fl^ Lyz2^cre+^*) (*n* = 4) and littermate controls (*n* = 4). (**H**) Wound healing curve for mice (*n* = 12) with loss of DIO DNMAML in CD4^+^ Th cells (DNMAM*L1^fl/fl^ C*D4^cre+^) compared with littermate controls (*n* = 10). (**I**) Analytical flow cytometry of cell suspensions 7 days after wounding between CD4^+^ Th cell–specific Notch-deficient diabetic (DIO) mice (DIO *DNMAML^fl/fl^ CD4^Cre+^)* (*n* = 5) and littermate controls (*n* = 5). Tregs identified as Lin^–^/CD3^+^/CD4^+^/CD25^+^/CD127^–^/FoxP3^+^ and Th17 cells identified as Lin^–^CD3^+^/CD4^+^/RORγ^+^. TF, total fluorescence. *P < 0.05, **P < 0.01, ***P < 0.001. Data are presented as the mean ± SEM. All data are representative of 2–4 independent experiments. **E** and **H** was analyzed with 2-way ANOVA.

Notch signaling plays an important role in regulating CD4^+^ Th cell phenotypes in various disease states; however, Notch signaling is highly context specific, and its role in CD4^+^ Th cells in wound repair has not been defined. Since our data suggest that Notch levels are altered in human T2D wounds and murine diabetic models, we sought to understand its role in physiologic wound healing. To achieve this end, we examined wound repair in our Notch signaling–deficient mice (7, 45). This was achieved with a cassette encoding the Notch inhibitor Dominant negative mastermind like protein 1 (DNMAML) at the ROSA26 locus downstream of a floxed stop codon sequence that prevents transcription of DNMAML, crossed with CD4^+^-Cre mice on a C57BL/6 background compared with littermate control (42). Mice deficient in CD4^+^ Th cell Notch signaling (*DNMAML^fl/fl^ CD4^cre+^*) demonstrated decreased wound healing after injury compared with Notch competent littermate controls (Supplemental Figure 2). In order to examine CD4^+^ Th cell phenotypes in wounds from these mice after injury, intracellular flow cytometry was performed on wounds isolated on day 3, and it showed a decreased numbers of Tregs (CD4^+^CD25^+^CD127^–^FoxP3^+^) and Th17 cells (CD4^+^RORγ^+^), as a percentage of total CD4^+^ Th cells, in the mice lacking Notch signaling (*DNMAML^fl/fl^ CD4^cre+^*) compared with the Notch-competent littermate controls (Figure 3A). In agreement with this, wounds from *DNMAML^fl/fl^ CD4^cre+^* mice demonstrated decreased IL-17A on ELISA (Figure 3B). Taken together, these results suggest that inhibition of Notch signaling in CD4^+^ Th cells reduces Th cell differentiation and is detrimental to normal wound repair.

We chose to focus on examining Notch1, given the prior work in other inflammatory disease states and tissues linking *Notch1* activation and Th17/Treg differentiation (30, 33–35, 41, 46) and relative paucity of literature examining *Notch2*, *Notch3*, and *Notch4* (30, 32). In order to examine the role of specific Notch receptor–ligand interactions, mice with a CD4^+^ Th cell–specific deficiency in Notch1 receptor (*Notch1^fl/fl^ CD4^cre+^*) were wounded. Compared with littermate controls, *Notch1^fl/fl^ CD4^cre+^* decreased Tregs and Th17 cells (Figure 3C) and decreased IL-17A protein in wound tissue from these mice (Figure 3D). These results suggest that the NOTCH1 receptor plays a role in wound Th cell differentiation and inflammatory signaling following cutaneous tissue injury. Additionally, Notch signals activated in Th cells via other Notch receptors (Notch2, -3, and -4) do not attenuate or fully compensate for the loss of NOTCH1 in wound CD4^+^ Th cells.

In tissues, Notch receptors are activated by a host of Notch ligands; one such ligant specific to NOTCH1 is DLL4 (28, 37). Since it has been previously found that DLL4 enhances Th17-mediated responses in lung Mφs, we examined the role of DLL4 ligand in Mφs, the primary APC in wound tissue, on CD4^+^ T cell phenotypes in wounds and wound repair following injury (47). To determine if Mφs play a role as a source of Notch ligand signal for wound CD4^+^ Th cells, and if DLL4 is important for wound healing, mice with a myeloid cell–specific deficiency of DLL4 (*DLL4^fl/fl^ Lyz2^cre+^*) were assessed for healing after 4 mm punch biopsy as previously described (2). We found that mice with a myeloid cell–specific deficiency in DLL4 demonstrated an impaired rate of wound repair with over half of the mice failing to heal by day 10 (Figure 3E) as well as decreased Tregs and Th17 cells in wound tissue and decreased IL-17A protein in wound tissue (Figure 3, F and G). These results suggest that DLL4 plays a role in facilitating physiologic Th cell phenotypes and the DLL4-Notch receptor interaction is important for normal wound repair to occur.

Since both murine and human diabetic wounds have increased Notch receptors, Th17 cells, and IL-17A levels, we hypothesized that by inhibiting Notch signaling in diabetic wound Th cells, we could improve wound healing by reducing CD4^+^ differentiation to Th17 and therefore lowering IL-17A levels in wounds by restoring a homeostatic balance to Treg and Th17 phenotypes. Surprisingly, diabetic mice deficient in CD4^+^ Th cell Notch signaling (*DIO DNMAML^fl/fl^ CD4^cre+^*) demonstrated decreased wound repair after injury, with less than 50% of the wound area healed by day 7 (Figure 3H) compared with Notch-competent, diabetic littermate controls, which we have previously demonstrated have completed robust wound healing by this time point (2, 6, 7, 48). In addition, decreased numbers of Tregs (CD4^+^CD25^+^CD127^–^FoxP3^+^) and Th17 cells (CD3^+^CD4^+^RORγ^+^), as a percentage of total CD4^+^ Th cells, were seen in the wounds from diabetic mice lacking Notch signaling (*DIO* *DNMAML^fl/fl^ CD4^cre+^*) compared with the Notch-competent littermate controls (Figure 3I). Taken together, these data suggest that both Tregs and Th17 cells are necessary for physiologic wound repair and that Notch inhibition and the resultant decrease in Tregs and Th17 cells, mediated through Mφ DLL4, is harmful to the wound healing process. Despite supraphysiologic expression of Th17 cells and IL-17A in diabetic wounds, nonspecific inhibition of Notch signaling and the resultant decrease in Th17 cells did not improve diabetic wound healing. Given that we identified that Notch signaling is critical for Th cell differentiation in cutaneous wounds and necessary for wound repair where complete abrogation of this pathway impairs healing in both a normal and diabetic setting, we next examined the regulatory mechanisms that may control increased Notch-related gene expression in diabetic wound tissue as well as control wound CD4^+^ Th cell phenotypic differentiation in an effort to not block but skew differentiation away from the pathologic Th17 cells without reducing the necessary Tregs that are vital for tissue repair.

### MLL1 in wound CD4^+^ Th cells regulates Notch1 and Notch2 receptor expression and downstream Notch signaling.

**Figure 4:**
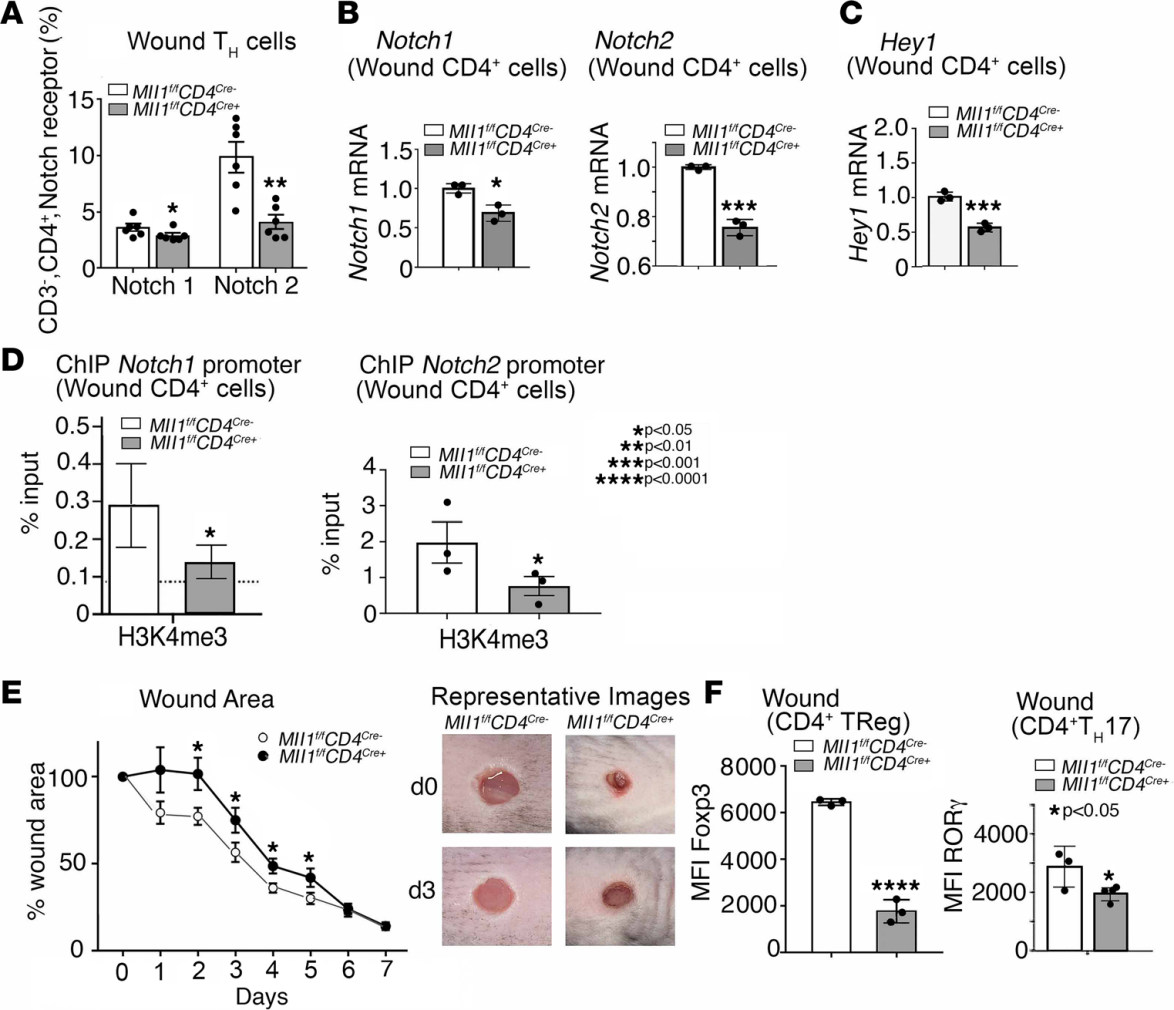
MLL1 in wound CD4^+^ Th cells regulates Notch1 and Notch2 receptor expression and downstream Notch signaling. (**A**) Analytical flow cytometry of wound cell suspensions 7 days after wounding between investigating Notch1 and Notch2 expression from mice with CD4^+^ Th cell–specific *Mll1*-deficient mice (*Mll1^fl/fl^ CD4^cre+^*) and littermate controls, (*n* = 6 mice/group, run in triplicate). (**B**) *Notch1* and *Notch2* expression in wound CD4^+^ Th cells 7 days after wounding between CD4^+^ Th cell–specific *Mll1* deficient mice (*Mll1^fl/fl^ CD4^cre+^*) and littermate controls (*n* = 6 mice/group, run in triplicate). (**C**) *Hey1* expression in wound CD4^+^ Th cells 7 days after wounding between CD4^+^ Th cell–specific *Mll1*-deficient mice (*Mll1^fl/fl^ CD4^cre+^*) and littermate controls (*n* = 6 mice/group, run in triplicate). (**D**) ChIP analysis of H3K4me3 on the promoters of *Notch1* and *Notch2* in CD4^+^ Th cells isolated from *Mll1^fl/fl^ CD4^cre+^* and littermate controls 3 days after wounding (*n* = 4 mice/group, run in triplicate). (**E**) Wound healing curve for mice (*n* = 12) with loss of *Mll1* in CD4^+^ Th cells (*Mll1^fl/fl^ CD4^cre+^*) compared with littermate controls (*n* = 10). (**F**) Analytical flow cytometry of wound cell suspensions 7 days after wounding between mice with CD4^+^ Th cell–specific Mll1-deficient mice (*Mll1^fl/fl^ CD4^cre+^*) (*n* = 3) and littermate controls (*n* = 4). Tregs identified as Lin^–^/CD3^+^/CD4^+^/CD25^+^/CD127^–^/FoxP3^+^, and TH17 cells identified as Lin^–^/CD3^+^/CD4^+^/RORγ^+^. *P < 0.05, **P < 0.01, ***P < 0.001, ****P < 0.0001. Data are presented as the mean ± SEM. All data are representative of 2–4 independent experiments. Data were first analyzed for normal distribution, and if data passed the normality test, 2-tailed Student’s *t* test was used. **E** was analyzed with 2-way ANOVA.

Since we identified that Notch signaling can regulate Th cell differentiation and Notch signaling is increased in diabetic wound CD4^+^ Th cells, we sought to examine the regulation of *Notch* receptor genes in wound CD4^+^ Th cells. Since our group and others have identified that CMEs can control immune cell differentiation and are relevant in wound repair, and since MLL1 has been shown to regulate Notch signaling in human cancer cell lines (7, 39), we examined the role of the histone methyltransferase MLL1 in wound CD4^+^ Th cells. Using our Mll1-floxed mice crossed to CD4^+^ Cre mice, we obtained mice with a CD4^+^ Th cell–specific deficiency in MLL1 (*Mll1^fl/fl^ CD4^cre+^*) and examined wound tissue for Notch-related genes and CD4^+^ T cell phenotypes. We performed flow cytometry on whole wounds isolated 3 days after wounding and found that the receptors Notch1 and Notch2 were decreased in MLL1-deficient CD4^+^ Th cells compared with their littermate controls (Figure 4A), whereas no differences were noted in the presence of receptors Notch3 and Notch4 (data not shown). Further, MLL1-deficient CD4^+^ Th cells isolated from wounded mice at multiple time points after wounding also demonstrated decreased *Notch1* and *Notch2* gene expression (Figure 4B), and Notch target gene *Hey1* (Figure 4C), compared with littermate controls. Next, in order to determine if MLL1 may regulate Notch 1 and 2 receptor expression, we isolated CD4^+^ Th cells from MLL1-deficient mice and performed a ChIP assay for H3K4me3, the activating chromatin mark set by MLL1. Increased H3K4me3 was seen on the promoters of *Notch1* and *Notch2* in wound CD4^+^ Th cells isolated from *MLL1^fl/fl^ CD4^cre–^* as compared with the *MLL1^fl/fl^ CD4^cre+^* (MLL1-deficient) CD4^+^ Th cells (Figure 4D). These data suggest that MLL1 regulates Notch1 and Notch2 receptor expression and that loss of MLL1 in wound CD4^+^ Th cells contributes to loss of Notch signaling/downstream Notch target gene expression in wound CD4^+^ Th cells.

Given that we found Notch receptors and downstream Notch target genes were impaired in wound CD4^+^ Th cells from *Mll1^fl/fl^ CD4^cre+^* mice, we examined wound repair in *Mll1^fl/fl^ CD4^cre+^* mice and found that, especially at early time points, wound healing was impaired in *Mll1^fl/fl^ CD4^cre+^* mice compared with littermate controls (Figure 4E). Whole wounds from these mice were then analyzed via intracellular flow cytometry for Th cell phenotypes. Wound CD4^+^ Th cells from MLL1-deficient mice displayed decreased Th17 cells, with a smaller decrease in Tregs (Figure 4F). In effect, these results demonstrate that MLL1 plays a role in dictating Notch signaling in wound CD4^+^ Th cells and, thus, CD4^+^ Th cell differentiation in wounds and that MLL1 in wound CD4^+^ T cells is important for tissue repair.

### MLL1 is increased in diabetic wound CD4^+^ T cells and skews wound CD4^+^ T cells toward Th17 by increasing Notch1 and Notch2 expression and by directly regulating RORγ.

**Figure 5:**
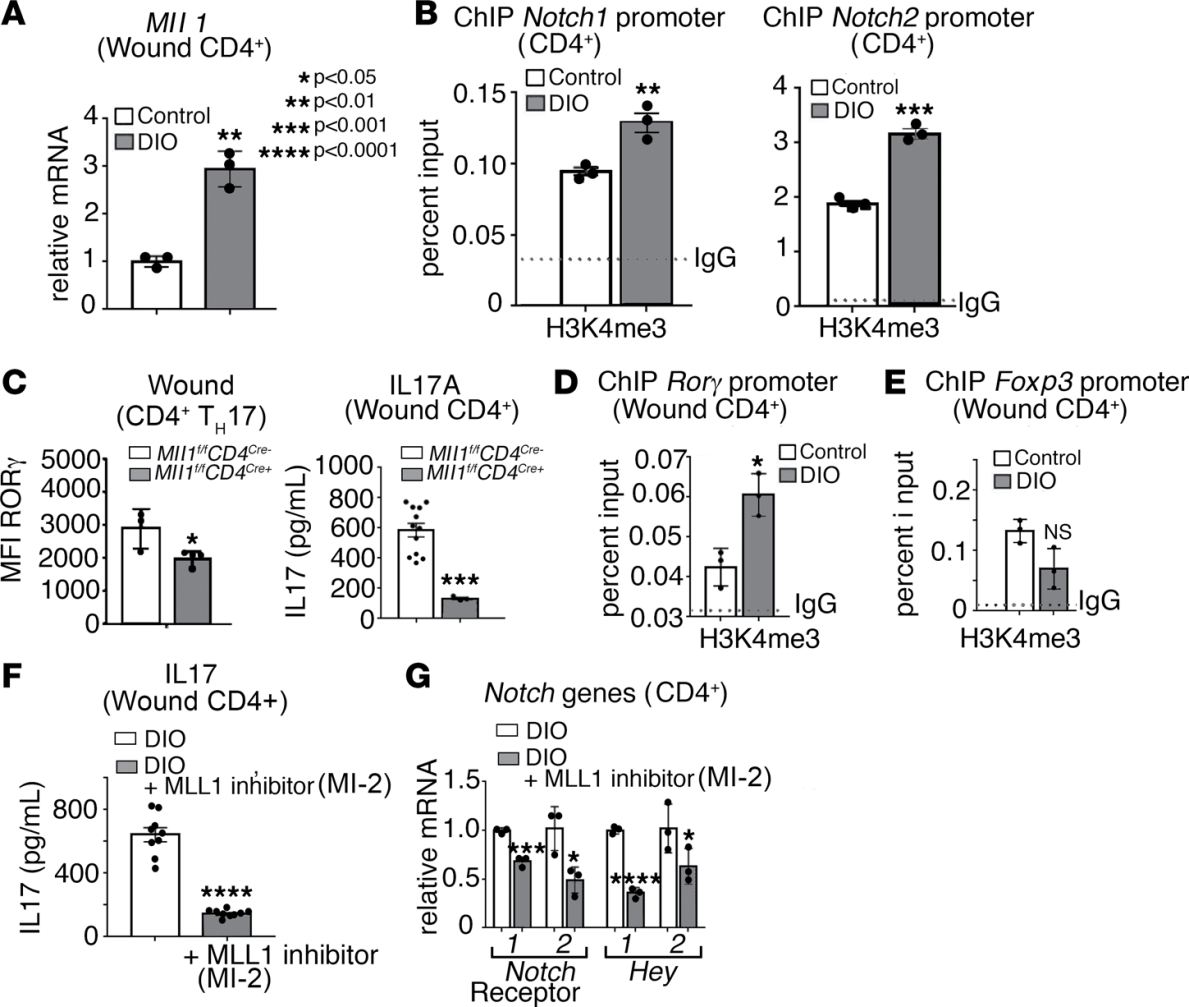
MLL1 is increased in diabetic wound CD4^+^ T cells and skews wound CD4^+^ T cells toward Th17 by increasing *Notch1* and *Notch2* expression and by directly regulating *RORγ*. (**A**) *Mll1* expression in wound CD4^+^ Th cells 7 days after wounding between ND and DIO mice (*n* = 6 mice/group, run in triplicate). (**B**) ChIP analysis of H3K4me3 on the promoters of *Notch1* and *Notch2* in CD4^+^ Th cells isolated from ND and DIO mice 3 days after wounding (*n* = 4 mice/group, run in triplicate). (**C**) Analytical flow cytometry of wound cell suspensions 3 days after wounding between DIO CD4^+^ Th cell–specific Mll1-deficient mice (*Mll1^fl/fl^ CD4^cre+^)* (*n* = 4) and littermate controls (*n* = 3) and IL-17 ELISA 5 days after wounding (*n* = 12 and *n* = 4). Th17 cells identified as Lin^–^/CD3^+^/CD4^+^/RORγ^+^. (**D**) ChIP analysis of H3K4me3 on the promoter of *RORγ* in ND and DIO CD4^+^ Th cells 7 days after wounding (*n* = 4 mice/group, run in triplicate). (**E**) ChIP analysis of H3K4me3 on the promoter of *Foxp3* in ND and DIO CD4^+^ Th cells 7 days after wounding (*n* = 4 mice/group, run in triplicate). (**F**) IL-17A ELISA of ex vivo DIO splenic CD4^+^ Th cells cultured 3 days under Th17 conditions with and without 1 μg/mL of the MLL1 inhibitor MI-2 (n = 9 mice/group). (**G**) *Notch1*, *Notch2*, *Hey1*, and *Hey2* gene expression of ex vivo DIO splenic CD4^+^ Th cells cultured 3 days under Th17 conditions with and without 1 μg/mL of the MLL1 inhibitor Mi-2 (*n* = 9 mice/group, run in triplicate).

Since MLL1 was shown to regulate Notch receptor expression and downstream Notch signaling in wound CD4^+^ Th cells and that we found that Notch signaling was increased in diabetic wound CD4^+^ Th cells, we examined the role of MLL1 in diabetic CD4^+^ T cells on Notch receptor expression and downstream Notch signaling. Murine wound CD4^+^ Th cells were isolated from wounds of DIO mice and control littermate controls and were examined for *Mll1* expression. Diabetic CD4^+^ Th cells demonstrated increased *Mll1* expression as compared with control CD4^+^ Th cells (Figure 5A). To determine if the increased *Mll1* expression resulted in a functional change in the CD4^+^ T cells, a ChIP assay was performed on DIO and control CD4^+^ T cells to examine MLL1-mediated H3K4me3 on the promoters of *Notch1* and *Notch2*. Increased H3K4me3 was found at the promoters of *Notch1* and *Notch2* in DIO CD4^+^ T cells (Figure 5B), suggesting that increased Notch receptors in DIO CD4^+^ Th cells may be in part due to upregulated MLL1.

Given that the diabetic wound CD4^+^ Th cells demonstrate a skewed differentiation toward the Th17 phenotype or away from the Treg phenotype (see Figure 1), we examined if genetic blockade of MLL1, specifically in CD4^+^ Th cells could reverse the increased Notch signaling in diabetic wound tissues to more physiologic levels and reduce Th17 differentiation in diabetic tissue. Wounds were isolated from mice subjected to a high-fat diet (HFD) for 12–16 weeks that were deficient in CD4^+^ Th cell–specific MLL1 (*DIO Mll1^fl/fl^ CD4^cre+^)* and littermate controls and were analyzed by intracellular flow cytometry. Wounds from mice with an MLL1 deficiency in CD4^+^ Th cells demonstrated decreased RORγ^+^ in Th17 cells as compared with littermate controls, suggesting that depletion of MLL1 in diabetic CD4^+^ Th cells can reduce Th17 differentiation. Phenotypically, IL-17A protein was also decreased by ELISA in CD4^+^ Th cells isolated from DIO *Mll1^fl/fl^ CD4^cre+^* mice as compared with littermate controls (Figure 5C).

**Figure 6:**
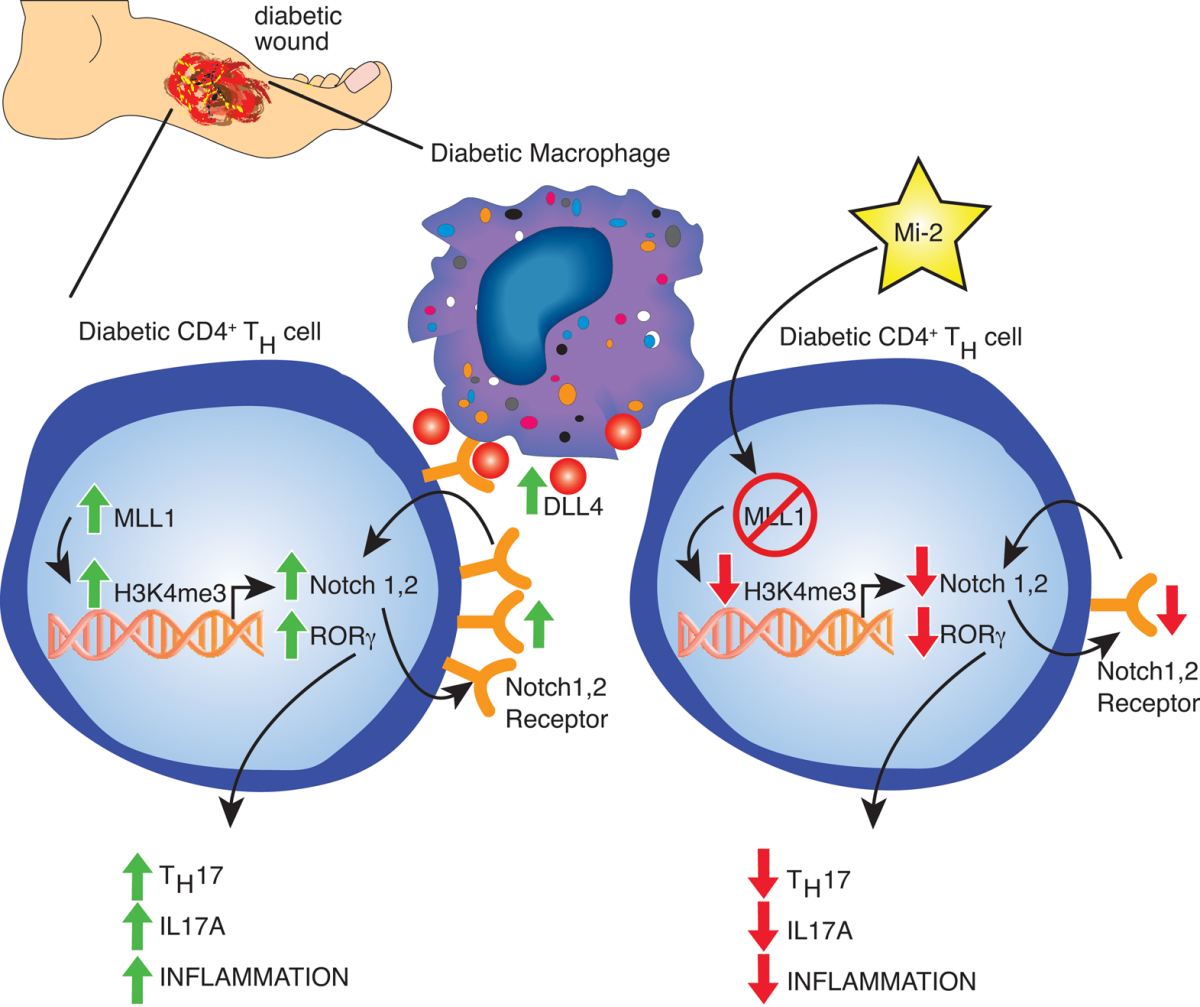
The histone methyltransferase Mixed-lineage-leukemia-1 (MLL1) drives CD4^+^ Th cell phenotype via Notch signaling in diabetic tissue repair

Given the reduction in Th17 cells with loss of MLL1, we also investigated whether MLL1 directly regulates expression of the Th17 associated transcription factor RORγ. We found increased H3K4me3 on the promoter of *RORγ* in diabetic CD4^+^ T cells as measured by ChIP (Figure 5D). We next sought to determine if MLL1 had an effect on FoxP3 expression. Despite increased *MLL1* in diabetic CD4^+^ Th cells, there was no increase in H3K4me3 on the promoter of *Foxp3* (Figure 5E). This differential regulation of MLL1 on *RORγ* and not *FoxP3* gene expression in the setting of diabetes may contribute to the imbalance and skewed differentiation of diabetic wound CD4^+^ Th cells seen toward a Th17 phenotype and away from the Treg phenotype in diabetic wound tissue. To examine the therapeutic potential of these findings, we isolated diabetic CD4^+^ T cells from wounds and treated them ex vivo with a small molecule inhibitor of MLL1, MI-2, and found this reduced IL-17A from the CD4^+^ T cells, as measured by ELISA (Figure 5F). Diabetic CD4^+^ Th cells treated with MI-2 also demonstrated decreased expression of Notch receptors *Notch1* and *Notch2*, in addition to the downstream Notch target genes *Hey1* and *Hey2* (Figure 5G). Taken together, these results suggest that increased MLL1 in diabetic CD4^+^ Th cells drives pathologic Th17 differentiation via addition of its activating H3K4me3 mark at *RORγ* promoter and indirectly through increased Notch receptor expression/signaling, and that inhibition of MLL1 with therapeutic targeting can reverse this process and reduce Th17- mediated inflammation in diabetic wounds (Figure 6).

## Discussion

In this study, we identified that CD4^+^ Th cell function is important for normal wound repair and that Notch signaling is crucial for physiologic CD4^+^ Th cell differentiation in wound repair. We showed that Notch signaling is upregulated in CD4^+^ Th cells in both human T2D as well murine models of diabetes, which correlates to increased Th cell differentiation to Th17 and supraphysiologic levels of IL-17A. Inhibition of Notch signaling through various methods reduced CD4^+^ Th cell differentiation to both Tregs and Th17cells and resulted in impaired wound healing. The epigenetic enzyme, MLL1, was found to be a key regulator of Notch receptors and downstream signaling and, thus, wound CD4^+^ Th cell differentiation. Increased MLL1 in diabetic wound CD4^+^ Th cells was found to drive Th17 differentiation through Notch signaling. Through treatment of diabetic CD4^+^ Th cells with a small molecule inhibitor targeting MLL1, pathologic IL-17A inflammation can be reversed. This promises an interesting avenue for further study into a cell-specific therapeutic target to improve wound healing in patients with diabetes.

It has been previously established that Th cell function is relevant in normal wound repair (22) and that depletion of CD4^+^ Th cells in WT mice causes substantial changes in wound inflammation and alters wound healing rates. However, the available literature is extremely limited and the regulation of Th cell function/phenotype in the setting of tissue injury has not been examined. Furthermore, although it is known that human and murine diabetic wounds display increased IL-17A and an imbalanced Th17/Treg ratio, the mechanisms responsible for this have not been identified. Although it is well known that Notch signaling can drive Th cell differentiation and function in other tissues and disease contexts, (25, 26, 49) the role of Notch signaling on CD4^+^ Th cell function following tissue injury in a normal or diabetic setting is unknown. Here, we show that disruptions of Notch signaling to wound Th cells via depletion of MAML, the Notch1 receptor, and adjacent DLL4 ligand on wound Mφs led to significant loss of Th cell differentiation toward Treg and Th17 phenotypes, resulting in impaired healing. This was somewhat expected given that both Th17 and Tregs are likely essential for proper tissue repair (7, 44). It was important to establish that loss of Notch signaling, and specifically DLL4/Notch1-mediated signaling, led to the loss of both Treg and Th17 populations in the wound, given how perturbations of Notch can be tissue and cell dependent (32, 33, 49). It was also valuable to confirm that disruption of Th17 cells due to Notch signaling depletion in the wound correlated strongly to loss of whole wound IL-17A protein; given that other lymphocytes, including neutrophils, have been shown to produce IL-17A (50) and could have maintained IL-17A levels (51). This is the first study to our knowledge to specifically examine Notch signaling in wound CD4^+^ Th cells following injury and to establish a link between Notch signaling and CD4^+^ Th cell differentiation in wounds.

Our group has a long history of investigating the pathophysiology behind prolonged pathologic inflammation in diabetic wound repair that contributes to delayed healing. Most of our prior work, as well as the work of many others in this field, has focused on the effect of myeloid cells as well as other structural cells in contributing to dysregulated inflammation and impaired immune responses in diabetic wound tissue that leads to impaired healing (2, 6, 44). Furthermore, although it is known that dysregulated Th cell–mediated inflammation drives other inflammation-associated complications in diabetes, such as retinopathy and nephropathy (20, 52, 53), the role of CD4^+^ Th cell differentiation on inflammation and wound repair has not been established. It has been previously found that IL-17A–mediated inflammation is pathologic in diabetic wound healing; however, the drivers of this excess IL-17A had not been known (23, 24, 54). The pathogenesis behind IL-17A–mediated inflammation in diabetes is incompletely understood, and here we examined if this increased IL-17A in diabetic wound tissue was driven in part by Notch-mediated increased Th17. Some prior research has suggested that Notch signaling is increased in diabetic wounds (7, 37) but has not specifically looked at wound CD4^+^ Th cells before. We found that Notch signaling was increased in human and murine diabetic wounds. Specifically in wound CD4^+^ Th cells, we found that NOTCH1 and NOTCH2 receptors were upregulated and subsequent downstream expression of Notch target genes (*Hes1*, *Hey1*, and *Hey2*) were also increased. Diabetic Th cells also were found to be exposed to increased Notch ligand, as human diabetic Mφs were found to express increased DLL4 ligand.

Given the increase in Notch signaling seen in diabetic wound Th cells, and prior work showing increased IL-17A in diabetic wounds (23, 24, 54) as well as increases in Th17 populations in other diabetic tissues (20, 52, 53), we expected that there would be increased Th17 cells seen in diabetic wounds. We did find this to be true in both human and murine wounds, and we also replicated the expected increase in IL-17A in diabetic wounds. However, despite our findings that loss of Notch signaling corresponds to loss of Tregs in nondiabetic wounds, we found that conversely, the increase in Notch signaling in diabetes did not increase Treg populations in diabetic wounds. In fact, we found that in diabetic wounds, Treg populations were decreased compared with nondiabetic controls. This is not entirely surprising given that prior literature showed an inverse relationship between Notch signaling on Tregs, depending on the context (32, 33, 49). This may be due in part to the extensive cross-regulation that occurs between Treg and Th17 cell populations (55). It may be that, in diabetic wound Th cells, the increase in Notch signaling drives such an increase in Th17 cells and IL-17A production that this, in turn, suppresses the Treg population. Given that Notch was increased in diabetic wound CD4^+^ Th cells, we examined whether inhibiting Notch signaling in diabetic wound Th cells could reduce the pathologic increase in Th17 cells and IL-17A, thus improving diabetic wound healing. However, we found that total Notch depletion via DNMAML caused a loss of both Treg and Th17 cell populations and actually impaired wound healing. Thus, we wanted to see if we could find a way to reduce Notch signaling but without depleting it to the point that it would interfere with physiologic Th cell function and differentiation.

To this end, we examined the regulation of CD4^+^ Th cell differentiation in the context of Notch in wound CD4^+^ Th cells. Limited prior research in oncology had shown that pathologic changes in Notch signaling were regulated by the histone methyltransferase MLL1 (39); however, this relationship had not been evaluated in Th cells or in wound tissue. Since similar CMEs are known to drive Th lineage via direct regulation of the key transcription factors *FoxP3* (Treg) and *RORγ* (Th17), we decided to test MLL1 as a potential regulator of Th cell differentiation in the setting of tissue repair (40). We also knew from our own prior work that MLL1 was increased in diabetes and contributed to pathologic inflammation in diabetic wound Mφs, making it a good candidate to examine in CD4^+^ Th cells (7). We showed that MLL1 does regulate Notch signaling in wound CD4^+^ Th cells and, thus, also the Treg/Th17 ratio. Depletion of MLL1 leads to loss of Notch receptors in wound CD4^+^ Th cells, via loss of protranscription H3k4me3 on the promoters of *Notch1* and *Notch2* genes. This correlated strongly with loss of Notch activity as measured by downstream *Hes1* and *Hey1*. We did identify that ex vivo treatment of CD4^+^ Th cells from wounds with an MLL1 inhibitor can reverse pathologic Th17 differentiation, making this a viable therapeutic target in diabetes to reverse Th17-mediated pathologic inflammation. Of note, our research specifically addresses Treg/Th17 ratios in diabetic skin but cannot comment on Th cell differentiation and ratios in lymph nodes. Further research is needed in the in vivo setting where a cell-specific targeting method will likely be needed, such as conjugation to CD3^+^ nanoparticles (56).

In summary, we found that Notch signaling regulates Th cell differentiation in wounds and is necessary for physiologic wound repair. Loss of Notch signaling decreases differentiation of both Tregs and Th17 cells and impairs healing. In diabetic wounds and specifically wound Th cells, Notch signaling is increased, with a corresponding increase in Th17 cells but not Tregs. Broadly inhibiting Notch signaling in diabetes did reduce Th17 populations but also depleted Tregs, and it did not rescue wound repair. Importantly, we identified that the histone methyltransferase MLL1 regulated Notch signaling by enhancing transcription of the *Notch1* and *Notch2* receptors. In diabetic Th cells, MLL1 was increased, which thereby increased Notch signaling and Th17 cell differentiation by directly driving a Th17 phenotype via activation of *RORγ* transcription. By blocking MLL1 activity in diabetic Th cells, we reversed the Th17 phenotype and reduced pathologic IL-17A levels, making MLL1 a viable therapeutic target.

## Methods

### Sex as a biological variable.

Sex was not considered as a biological variable in all reported human data. Only males were used in the murine studies, since female C57BL/6 mice do not exhibit the DIO phenotype when placed on an HFD.

### Mice.

All mice were maintained at the University of Michigan in the Unit for Laboratory and Animal Medicine. Mouse experiments were conducted with approval from the University of Michigan IACUC, and all regulatory and safety standards were strictly adhered to. C57BL/6 mice were obtained at 6–7 weeks of age from The Jackson Laboratory and were maintained in breeding pairs at the Unit for Laboratory Animal Medicine (ULAM) facilities. Mice with the *Dnmaml*, *Notch1*, or *Mll1* gene deleted in Th cells (*Dnmaml^fl/fl^ CD4^Cre+^*, *Notch1^fl/fl^ CD4^Cre+^*, *Mll1^fl/fl^ CD4^Cre+^*) were generated by mating floxed mice with *CD4^Cre^* mice (The Jackson Laboratory) (6, 45). Mll1 deletion efficacy in the *Mll1^fl/fl^ CD4^Cre+^* was 87% (data not shown). Mice with the DLL4 gene deleted in myeloid cells (*Dll4^fl/fl^ Lyz2^Cre+^*) were generated by mating *Dll4^fl/fl^* mice with *Lyz2^Cre^* mice. Animals were housed in a barrier facility on a 14-hour light/10-hour dark cycle (ambient temperature of 22°C) with free access to water, food (Laboratory Rodent Diet 5001), and bedding (Anderson Lab Bedding Bed-o’Cobs combo). The number of animals per group in all the experiments was determined on the basis of prior literature, power calculation, and experience from our previous studies to ensure sufficient sample sizes to allow the detection of statistically significant differences. No animals were excluded from analysis. The number of replicates for each experiment is labeled in all figures or legends. Animal studies were approved by the ethics committees of the University of Michigan IACUC.

### Murine model of diabetes.

To induce a prediabetic state, male C57BL/6 mice were maintained on a standard HFD (60% kcal saturated fat, 20% protein, 20% carbohydrate; Research Diets Inc.) for 12–18 weeks to induce the DIO model of T2D as previously described (2). After the appropriate period, HFD-fed (DIO) mice developed obesity and insulin resistance with fasting blood sugars in the mid-200 mg/dL and elevated insulin levels. All animals underwent procedures at 20–32 weeks of age with approval from the University of Michigan IACUC. For these experiments, only male mice were used because female mice do not develop DIO. Number of mice used per experiment can be found in the figure legend of each corresponding experiment.

### Murine wound healing model.

Mice were anesthetized, dorsal hair was removed with Veet (Reckitt Benckiser) and rinsed with sterile water, and 2 full-thickness back wounds were created by 6 mm punch biopsy with or without wound splinting. The initial wound surface area was recorded, and digital photographs were obtained daily using an 8 mm iPad camera as previously described (57). Photographs contained an internal scale to allow for standard calibration. The wound area was quantified using ImageJ software (NIH) and calculated as a percentage of initial wound area.

### MACS of murine CD4^+^ Th cells.

Briefly, following tissue morcellation, wounds were digested in a suspension of liberase (50 mg/mL; Sigma-Aldrich, 5101020001) and DNase I (20 U/mL; Sigma-Aldrich, 9003-98-9) solution at 3 °C for 30 minutes. RPMI + FBS were then added to stop the reaction, and the cells were filtered through a 100 μm filter and subsequently run through a CD4^+^ MACS Selection Kit (Stemcell Technologies, 18952), achieving a 98.4% purity measured by FACS. For splenocytes, no digestion was performed and the morcellated samples were simply filtered through a 40 μm filter before running MACS. For naive CD4^+^ Th cells, a negative selection kit was utilized (Stemcell Technologies, 19765).

### Ex vivo naive CD4^+^ culture and treatment with MLL1 inhibitor.

Wound cell suspensions underwent MACS for naive CD4^+^ Th cells, and subsequent cell suspensions were plated in H-2 media (RPMI + 10% FBS + 1 P/S + 1:1,000 BME) with Th17 conditions (1 μg/mL of CD28, 2.5 μg/mL of IFN-γ, 2.5 μg/mL of IL-4, 20 ng/mL of IL-6, 2 ng/mL of TGF-β) on 24-well plates precoated with CD3ε (3 μg/mL for 3 hours). Experimental groups were also given 1 μg/mL of MLL1 inhibitor MI-2 (444825, MilliporeSigma). Cells and cell-free supernatants were harvested after 3 days of treatment for analysis of RNA expression via qPCR and IL-17A protein concentration via ELISA.

### Flow cytometry.

Wound cells were first stained with a LIVE/DEAD Fixable Yellow Dead Cell Stain Kit (Molecular Probes, Invitrogen, L34959; 1:1,000 dilution) and then washed 2 times with cold PBS. Cells were then resuspended in flow buffer (500 mL PBS, 0.5 g BSA, 5 mL 2% NaN_3_ in water, and 5 mL of 1M Hepes Buffer), and Fc-receptors were blocked with anti-CD16/32 (BioXCell, CUS-HB-197, 1:200 dilution) prior to surface staining. Following surface staining, cells were washed twice. Next, cells were either washed and acquired for surface-only flow cytometry, or they were fixed with 2% formaldehyde and then washed/permeabilized with BD perm/wash buffer (BD Biosciences, 00-8333-56) for intracellular flow cytometry. Antibodies utilized for flow cytometry are as follows: H3k4me3 (Abcam, 015495-2), CD3 APC (Invitrogen, 17-0031-82, 2612978), CD4 (BioLegend, 100547, B399084), CD25 (BioLegend, 102051, B396255), FoxP3 (BioLegend, 126404, B375703), RORγ (Invitrogen , 2-6988-82, 2504535), CD19 (BioLegend, 115506, B256058), Gr1 (BioLegend, 108406, B236798), and CD8 (BioLegend, 100705, B393336).

After washing, samples were then acquired on a 3-Laser Novocyte Flow Cytometer (Acea Biosciences). Data were analyzed using FlowJo software version 10.0 (Tree Star Inc.) and compiled using Prism software 9.2 (GraphPad). Tregs were initially identified in diabetic and nondiabetic wounds as CD3^+^CD4^+^CD25^+^FoxP3^+^ cells; however, to improve the specificity of our gating strategy, we altered our identification of Tregs to include cells that are CD3^+^CD4^+^CD25^+^CD127^–^FoxP3^+^, in agreement with the most recent scientific description of specific Treg populations (58, 59).To verify gating and purity, all populations were routinely back-gated.

### RNA isolation.

Total RNA extraction was performed with Trizol (Invitrogen, Thermo Fisher Scientific) using the manufacturer directions. RNA was extracted using chloroform, isopropanol, and ethanol. iScript (Bio-Rad) or Superscript III Reverse Transcriptase (Thermo Fisher Scientific) kits were used to synthesize cDNA from extracted RNA. We used cDNA primers for *Notch1* (*Mm00627185_m1*), *Notch2* (*Mm00803077_m1*), *Notch3* (*Mm01345646_m1*), *Notch4* (*Mm00440525_m1*), *Hey1* (*Mm00468865_m1*), *Hey2* (*Mm01180513_m1*), *Hes1* (*Mm01342805_m1*), *Dll4* (*Hs00184092_m1 and Mm00444619_m1*), *Mll1* (*Mm01179235_m1*) and 18s or GAPDH as the internal control. Data were analyzed relative to 18S ribosomal RNA or GAPDH (2^–ΔCt^). All samples were assayed in triplicate. The Ct values were used to plot a standard curve. Data are representative of 2–3 independent experiments were compiled in Microsoft Excel and presented using Prism software (GraphPad).

### Inflammatory cytokine ELISA.

Either whole wound homogenates or ex vivo isolated CD4^+^ Th cells had IL-17A levels measured by ELISA kits for IL-17A (R&D systems) per the manufacturer’s protocol.

### Western blot.

Cell suspensions were lysed in RIPA buffer (MilliporeSigma) and standardized for protein concentrations using a Bradford protein assay (Bio-Rad) to generate a standard curve. Equal amounts of protein were then loaded onto to 4%–12% SDS gel electrophoresis under reducing conditions. Protein bands were then transferred to polyvinylidene difluoride (PVDF) membranes and probed with primary antibodies anti–mouse MLL1 (14197, Cell Signaling Technology) and anti–mouse NICD (Val1744 D3B8, Cell Signaling Technology) at 4°C for 12 hours. All primary antibodies were diluted 1:500 in 5% BSA in 0.1 % Tween Tris-buffered saline (TBS-T) solution. PVDF membranes were then washed and incubated with HRP-labeled secondary antibody (Invitrogen, PI32460) for 1 hour at room temperature and visualized with chemiluminescence (ChemiDoc XRS+; Bio-Rad). Blot images were analyzed using NIH ImageJ software to obtain sample densitometry readings normalized to β-actin.

### ChIP assay.

ChIP assay was performed as described previously (2). Briefly, cells were fixed in 1% paraformaldehyde and lysed and sonicated using a Bioruptor Pico (Diagenode) to generate 300–500 bp fragments. Samples were then incubated overnight in anti-H3K4me3 antibody (ab8850, Abcam) or isotype control (rabbit polyclonal IgG; ab171870, Abcam) in parallel followed by addition of protein A-Sepharose beads (Thermo Fisher Scientific). Beads were washed and bound; DNA was eluted and purified using phenol/chloroform/isoamyl alcohol extraction followed by ethanol precipitation. H3K4me3 deposition was measured by qPCR using 2X SYBR PCR mix (Thermo Fisher Scientific) and primers targeting NF-κB binding sites in the *Notch1*, *Notch2*, *Rorγ*, and *Foxp3* promoters. Primers were designed using the Ensembl genome browser to search the Notch1, Notch2, Rorγ, and Foxp3 promoters for NF-κB within the promoter region, and then NCBI Primer-BLAST was used to design primers that flank this site. The following primers were used to amplify DNA in samples: Notch1: 5′ – AATGGAGGGAGGTGCGAAGT – 3′ and 5′ – AAGGCTCCGCTGCAGACA – 3′; Notch2: 5′ – CCGCTGTCCCCCTCAGT – 3′ and 5′ – AGCGTTGGGCTGCAACA – 3′; Rorγ: 5′ – CCCCTCACCTCTCAATTTGC – 3′ and 5′ – GCTTCTAGATGCTTCCCATACTTCTG – 3′; and Foxp3: 5′ – GGGCACTCAGCACAAACATGATG – 3′ and 5′ – GAGGCTTCCTTCTGCCCAAAC – 3′.

### Human monocyte isolation.

For human monocyte isolation, peripheral blood was collected from patients in the University of Michigan Peripheral Artery Disease (UM PAD) clinic with diabetes (*n* = 9) and without diabetes (*n* = 8), and buffy coat was isolated via centrifugation (800*g*). Cell suspensions were then treated with anti–human CD14 microbeads (EasySep Human CD14 Positive Selection Kit, Stemcell Technologies) and purified by MACS as described above.

### Human scRNA-Seq patient population.

Within the samples from patients with diabetes, for scRNA-Seq, the average age was 60 years, with all patients having diabetes, hypertension, hyperlipidemia, and coronary artery disease. Within the samples from patients without diabetes, the average age was 70 years, with 50% of patients having hypertension, hyperlipidemia, and coronary artery disease.

### scRNA-Seq and bulk RNA-Seq.

Generation of single-cell suspensions for scRNA-Seq was performed in the following manner. Following informed consent from patients and in accordance with University of Michigan IRB (HUM00098915), skin was harvested via punch biopsy from diabetic and nondiabetic control wounds. Skin samples from human wounds was obtained by 6 mm punch biopsy. Samples were incubated overnight in 0.4% dispase (Invitrogen) in HBSS (Thermo Fisher Scientific) at 4°C. Epidermis and dermis were separated. Epidermis was digested in 0.25% Trypsin-EDTA (Thermo Fisher Scientific) with 10U/mL DNase I (Thermo Fisher Scientific) for 1 hour at 37°C, quenched with FBS (Atlanta Biologicals), and strained through a 70 μM mesh. Dermis was minced, digested in 0.2% Collagenase II (Invitrogen) and 0.2% Collagenase V (MilliporeSigma) in plain medium for 1.5 hours at 37°C, and strained through a 70 μM mesh. Epidermal and dermal cells were combined in a 1:2 ratio, and libraries were constructed by the University of Michigan Advanced Genomics Core on the 10X Chromium system with chemistry v2 and v3. Libraries were then sequenced on the Illumina NovaSeq 6000 sequencer to generate 150 bp paired-end reads. Data processing, including quality control, read alignment (hg38), and gene quantification was conducted using the 10X Cell Ranger software. The samples were then merged into a single-expression matrix using the cellranger aggr pipeline. For bulk RNA-Seq and scRNA-Seq data accession, the numbers include GSE154556 and GSE154557 (Gene Expression Omnibus [GEO]).

For bulk RNA-Seq, we computed the principal components (PCs) using the topmost 2,000 variable genes, and we calculated the Uniform Manifold Approximation and Projection (UMAP) with the top 30 topmost variable PC after batch effect correction with harmony. Cells were then clustered using shared nearest neighbor modularity optimization–based clustering, and marker genes were identified with FindAllMarkers. Cell typing was conducted by comparing marker genes to curated cell-type signature genes. NOTCH signaling was identified to be one of the most significant pathways enriched among the upregulated genes in our full control sex-matched analysis (*P* = 5.78 × 10^–3^ based on hypergeometric test). We have highlighted all the genes that were consistently altered in diabetic skin compared with controls belonging to the Notch signaling pathway.

### Cell clustering and cell type annotation.

The R package Seurat (v3.1.2) (60) was used to cluster the cells in the merged matrix. Cells with less than 500 transcripts or 100 genes, or more than 10% of mitochondrial expression, were first filtered out as low-quality cells. The NormalizeData function was used to normalize the expression level for each cell with default parameters. The FindVariableFeatures function was used to select variable genes with default parameters. The FindIntegrationAnchors and IntegrateData functions were used to integrate the samples prepared using different 10X Chromium chemistries. The ScaleData function was used to scale and center the counts in the data set. Principal component analysis (PCA) was performed on the variable genes, and the first 20 PCs were used for cell clustering and UMAP dimensional reduction. The clusters were obtained using the FindNeighbors and FindClusters functions with the resolution set to 0.5. The cluster marker genes were found using the FindAllMarkers function. The cell types were annotated by overlapping the cluster markers with the canonical cell type signature genes. To calculate the disease composition based on cell type, the number of cells for each cell type from each disease condition was counted. The counts were then divided by the total number of cells for each disease condition and scaled to 100% for each cell type. Differential expression analysis was carried out using the FindMarkers function.

### Cell type subclustering.

Subclustering was performed on the abundant cell types. The same functions described above were used to obtain the subclusters. Subclusters that were defined exclusively by mitochondrial gene expression, indicating low quality, were removed from further analysis. The subtypes were annotated by overlapping the marker genes for the subclusters with the canonical subtype signature genes.

### Statistics.

GraphPad Prism software (RRID:SCR_002798) version 9.2 was used to analyze the data. Data were analyzed for normal distribution, and then statistical significance between multiple groups was determined using a 1-way ANOVA followed by Newman-Keuls post hoc test. For all single-group comparisons, if data passed normality test, we used a 2-tailed Student’s *t* test. Otherwise, data were analyzed using the Mann–Whitney *U* test. For repeated measures, data were analyzed by 2-way ANOVA. All data are representative of at least 2 independent experiments as detailed in the figure legends. A *P* value of less than or equal to 0.05 was significant.

### Study approval.

All experiments using human samples were approved by the IRB at the University of Michigan (IRB HUM00098915) and were conducted in accordance with the principles in the Declaration of Helsinki. All mice used were on a C57BL/6 background. Mice were housed at the University of Michigan Biomedical Sciences and Research Building in the ULAM, a pathogen-free animal facility. Mouse experiments were conducted with approval from the University of Michigan IACUC (protocol no. PRO00009811), and all regulatory and safety standards were strictly adhered to.

### Data availability.

For bulk RNA-Seq and scRNA-Seq data accession, the numbers include GSE154556 and GSE154557 (GEO). Values for all data points in graphs are reported in the Supporting Data Values file. 

## Author contributions

WJM and TMB contributed equally to this manuscript as co–first authors; WJM had a more prominent role in experimental design and is therefore listed first. FMD and KAG contributed equally to this manuscript as co–senior authors; KAG had a more prominent role in experimental design and is therefore listed last. WJM, KAG, KDM, TMB, SJW, RB, SLK, JEG, JS, ECB, and FMD designed the experiments. WJM, KDM, JS, ECB, ADJ, JYM, RB, COA, PM, and ECB performed experiments. TMB, RW, KDM, COA, SLK, RB, SJW, JEG, ADJ, JYM, WJM, TMB, and ECB analyzed data. WJM, TMB, and KAG wrote the manuscript. All authors reviewed, edited, and accepted the manuscript in its final form.

## Supplementary Material

Supplemental data

Supporting data values

## Version 1 09/09/2024

In-Press Preview

## Version 2 10/08/2024

Electronic publication

## Version 3 10/09/2024

Rachael Bogle's name was corrected
