# Supplementary material for: The histone methyltransferase Mixed-lineage-leukemia-1 drives T cell phenotype via Notch signaling in diabetic tissue repair
Source: JCI Insight. 2024 Oct 8;9(19):e179012. doi: 10.1172/jci.insight.179012 (PMC11463913; doi:10.1172/jci.insight.179012)
Supplement: Supplemental data [file jciinsight-9-179012-s244.pdf]

Supplemental Figures:

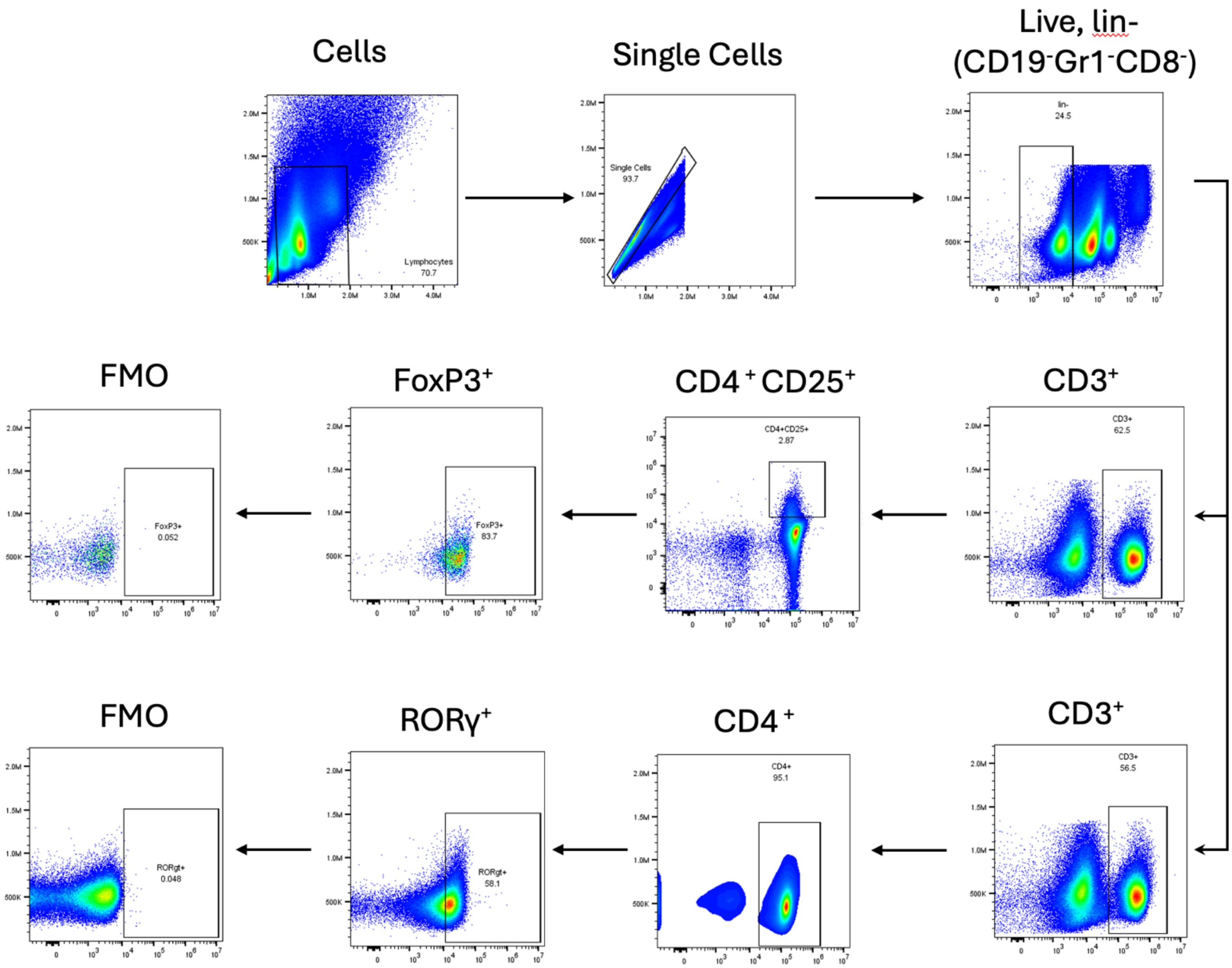

Supplemental Figure 1: Flow cytometry gating strategy for wound and splenic T cells.

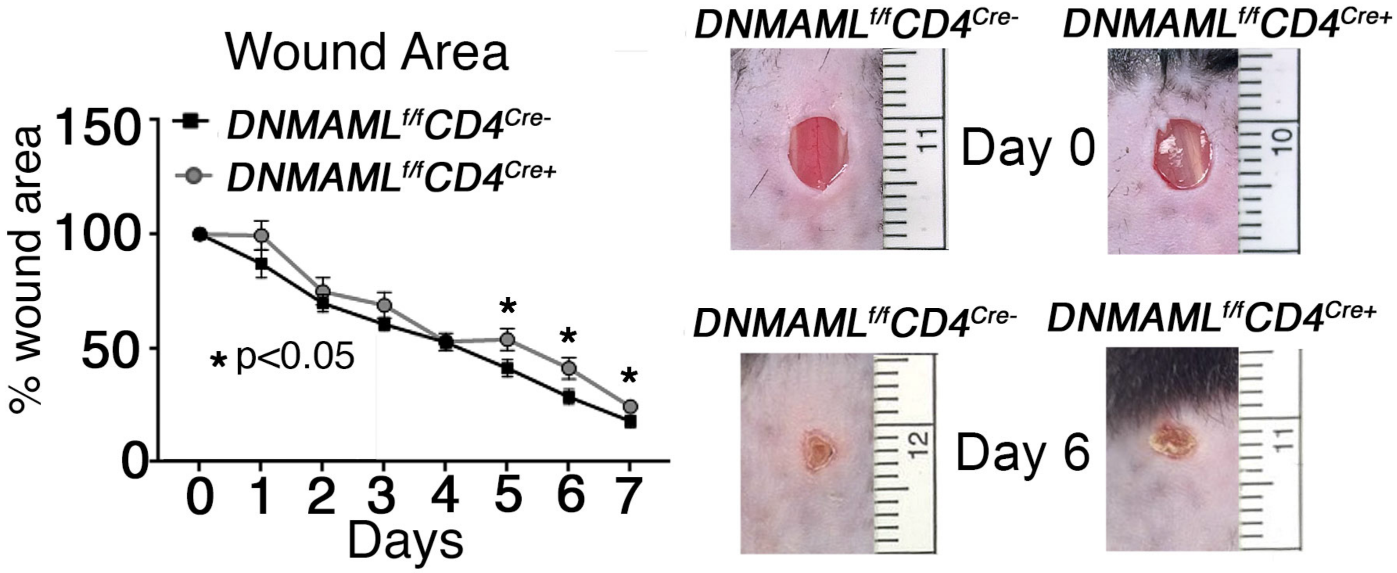

Supplemental Figure 2: Wound healing curve for mice (n=8) with loss of Notch signaling in $CD4^+$ cells (*DNMAML$^{f/f}$CD4^{cre+}*) compared to littermate controls (n=7).
